# PCaseek: ultraspecific urinary tumor DNA detection using deep learning for prostate cancer diagnosis and Gleason grading

**DOI:** 10.1038/s41421-024-00710-y

**Published:** 2024-09-03

**Authors:** Gaojie Li, Ye Wang, Ying Wang, Baojun Wang, Yuan Liang, Ping Wang, Yudan He, Xiaoshan Hu, Guojun Liu, Zhentao Lei, Bao Zhang, Yue Shi, Xu Gao, Xu Zhang, Weimin Ci

**Affiliations:** 1 China National Center for Bioinformation, Beijing, China; 2 Beijing Institute of Genomics, Chinese Academy of Sciences, Beijing, China; 3 University of Chinese Academy of Sciences, Beijing, China; 4 Department of Urology, Chinese PLA General Hospital, Beijing, China; 5 Department of Urology, Aerospace Center Hospital, Beijing, China; 6 Department of Urology, Changhai Hospital, Naval Military Medical University, Shanghai, China

**Keywords:** Prostate cancer, Tumour biomarkers

Dear Editor,

Prostate cancer (PCa) is the 2nd most common cancer, with an estimated 1.4 million new cases worldwide in 2020^1,2^. The diagnosis of PCa depends on prostate tissue samples obtained during biopsy or surgery. Treatment for PCa is typically guided by key clinicopathological factors, including serum prostate-specific antigen (PSA) levels, clinical staging, biopsy Gleason Score (GS), patient age, and comorbidities^3^. Serum PSA screening has been widely used to diagnose PCa. However, the PSA test lacks specificity, particularly within the gray zone (2‒10 ng/mL)^4^. Most experts recognize that PSA testing increases the risk of overdetecting otherwise indolent diseases and the consequential risk of overtreatment, which may lead to patient anxiety and treatment-related morbidities^5^. Thus, an accurate test to diagnose and differentiate moderately/highly aggressive PCa (International Society of Urological Pathology (ISUP) grade ≥ 3, High Grade) from less/slightly aggressive PCa (ISUP grade ≤ 2, Low Grade) before a biopsy is urgently needed.

Recent studies, including ours, have shown that detecting cancer signals, including copy number alterations, fragmentation patterns, and DNA methylation profiles, in urinary DNA high-throughput sequencing data is emerging as a novel noninvasive cancer detection method for urological cancers^6–8^. The major challenge of these approaches is how to identify useful biomarkers from the tiny amount of tumor DNA among the total urinary DNA, especially for PCa patients. It is widely accepted that DNA methylation detection using liquid biopsies is a promising approach not only for early cancer diagnosis but also for prognostic assessment. In addition, DNA methylation patterns are pervasive, which means that the same methylation patterns (methylated or unmethylated) tend to spread throughout a genome region. This feature inspired a number of recent approaches that use DNA methylation patterns for cancer diagnosis^9,10^. A recently proposed deep learning model named DISMIR can achieve ultrasensitive and robust cancer detection by integrating DNA sequence and methylation information of individual sequencing reads from plasma cfDNA whole-genome bisulfite sequencing (WGBS) data^11^. Here, we constructed two deep learning models, PCaseek-D and PCaseek-G, to **d**iagnose and perform Gleason **g**rading using urinary DNA WGBS data from PCa patients before a biopsy.

We started by randomly selecting 25 sets of our previously published WGBS data (high Gleason score (HGS) ≥ 4 + 3, *n* = 15; low Gleason score (LGS) ≤ 3 + 4, *n* = 10) from tumor tissues and matched adjacent normal tissues of PCa patients (> 30X genome coverage of each sample)^12^. We also included 171 in-house-generated WGBS data (3X‒5X genome coverage of each sample) from whole urine DNA of PCa patients (*n* = 87) and benign prostatic diseases and healthy individuals (*n* = 84). The training cohort contains 25 sets of WGBS data from tumor tissues and matched adjacent normal tissues of PCa patients, combined WGBS data of urinary DNA from 40 randomly chosen PCa patients (HGS, *n* = 30; LGS, *n* = 10), and combined WGBS data of urinary DNA from 40 randomly chosen benign prostatic disease individuals (*n* = 31) and healthy individuals (*n* = 9) (Supplementary Fig. S1a, b and Tables S1, S2). The remaining 91 urine samples collected from 47 PCa patients and 44 benign patients were used as the validation cohort (Supplementary Fig. S1d and Table S2). After the model training was completed, we subsequently collected 56 urine samples, including 32 cases from PCa patients and 24 cases from benign prostatic disease, to serve as an external independent validation set for assessing the model’s performance (Supplementary Table S2).

Before constructing the model, we first obtained and compared two types of differentially methylated regions (DMRs) on the basis of two different signatures in the training cohort (Supplementary Fig. S2). We selected DMRs with **m**ean methylation levels that were significantly different between tumor tissues and matched adjacent normal tissues as well as combined urine samples of noncancer individuals, and referred to these regions as ‘mDMRs’ (see Methods for details). Then, we introduced a new feature named ‘PCa-specific pDMR’. The region is called pDMR if the **p**roportion of hypo/hyper-methylated tumor-derived reads within DMRs is higher than that of adjacent normal tissue and noncancer urinary DNA (see Methods for details and Supplementary Fig. S3a, b). We clustered 25 paired WGBS datasets, including tumor tissues and matched normal tissue datasets, using pDMRs and mDMRs. Unsupervised clustering based on pDMRs led to a better performance than that based on mDMRs, indicating that aberrant DNA methylation signals at the resolution of single sequencing reads enabled the ultrasensitive detection of a tiny amount of tumor DNA (Fig. 1a, d; Supplementary Fig. S3c‒h and Table S3). Furthermore, the distribution of *α*-values of reads in four representative PCa-specific pDMRs was significantly different between PCa tissues and adjacent normal tissues, as well as between urine samples from PCa patients and noncancer individuals (Supplementary Fig. S3i, j). Therefore, for Model I, PCaseek-D, we used multimodal information that included the methylation states and the surrounding DNA sequences of individual reads within PCa-specific pDMRs and trained a deep learning model, PCaseek-D, on the training cohort (Supplementary Fig. S1c). Considering that the number of markers can influence the model performance, the value of the threshold determines how many pDMRs are identified. We observed the relationship between the threshold and the accuracy of the model in the validation cohort and set the threshold as 2000 hypomethylated PCa-specific pDMRs (Fig. 1e; Supplementary Fig. S3k). The estimated ratio of tumor-derived reads by PCaseek-D was defined as the PCaseek-D score. The PCaseek-D score can significantly differentiate PCa patients from noncancer individuals using in-house-generated 171 sets of urinary DNA WGBS data, which are fully independent of Gleason grading, and patient age, but not of PSA level (Fig. 1f‒h; Supplementary Fig. S4a). Then, we use the Youden index to determine the optimal cutoff for the model score, which is a common summary statistic of the receiver operating characteristic (ROC) curve for evaluation the effectiveness of certain biomarkers (see Methods for details)^13^. Notably, PCaseek-D achieved area under the ROC curves (AUCs) of 0.98 and 0.97 in diagnosing PCa on the training and validation cohorts, respectively (Fig. 1i, j; Supplementary Fig. S4b, c). Furthermore, PCaseek-D exhibited excellent diagnostic ability in the independent validation cohort with high specificity (92%) and acceptable sensitivity (72%) (Supplementary Fig. S4d). Additionally, PCaseek-D maintained high precision at ultralow sequencing depths (0.3X‒0.5X) on the validation cohort (Fig. 1k; Supplementary Fig. S5). More importantly, PCaseek-D outperformed the serum total PSA test (tPSA) and other related parameters, such as free-to-total PSA (%fPSA) and prostate-specific antigen density (PSAD), on the validation cohort (Fig. 1l‒n; Supplementary Fig. S6a, b). Moreover, PCaseek-D showed great performance for PCa with PSA levels in the gray zone of 2‒10 ng/mL (Fig. 1o; Supplementary Fig. S6c).

**Fig. 1:**
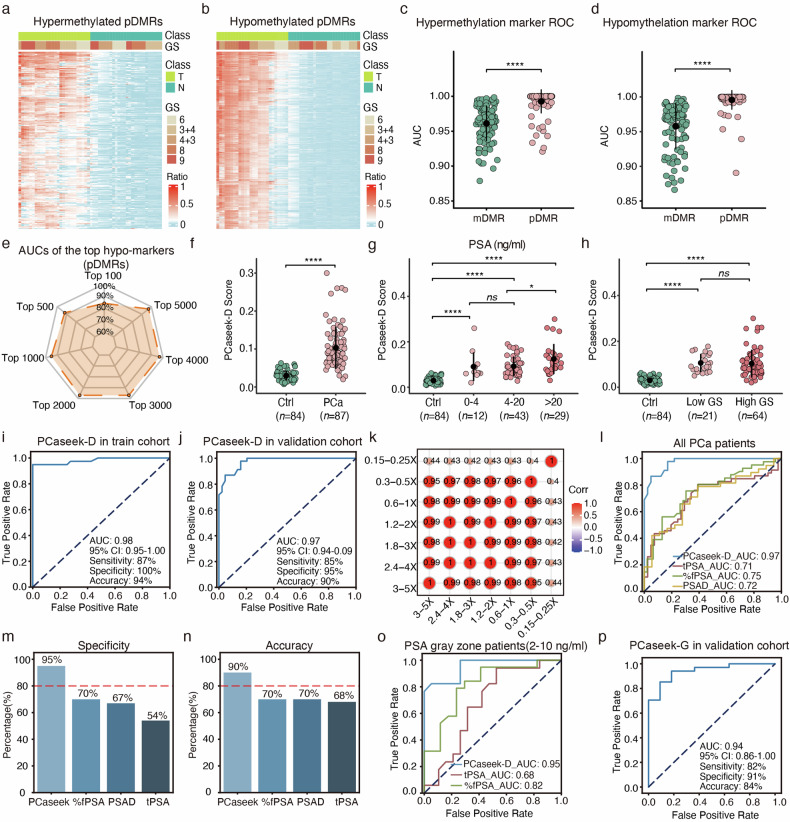
The clinical prediction using the PCaseek-D and PCaseek-G models. **a, b** Unsupervised hierarchical clustering of the proportion of the top 10% of PCa-specific hyper (**a**)/hypo (**b**) pDMRs, ranked by differential methylation values, between PCa tissues (*n* = 25) and adjacent normal tissues (*n* = 25). **c, d** Scatterplots of AUC values for the top 100 PCa-specific hyper (**c**) and hypo (**d**) pDMRs and mDMRs, with each point representing the classification performance of a pDMR or mDMR region between PCa tissues (*n* = 25) and adjacent normal tissues (*n* = 25). **e** Radar chart of the relationship between the threshold for the number of selected hypomethylated PCa-specific pDMRs and the accuracy of the PCaseek-D model on the validation cohort (*n*_tumor_ = 47, *n*_benign_ = 44). **f–h** Scatterplots of the distribution of PCaseek-D scores for the control group and tumor group (**f**) with different PSA levels (**g**) and with low GS (Gleason score ≤ 3 + 4) and high GS (Gleason score ≥ 4 + 3) (**h**). The control group consists of healthy individuals and benign prostatic disease patients. **i, j** ROC curves of the PCaseek-D model on the training cohort (**i**) and the validation cohort (**j**). **k** Correlation matrix plot of PCaseek-D scores at various sequencing depths on the validation cohort. **l** The ROC curve and corresponding AUC values of PCaseek-D, tPSA, %fPSA, and PSAD on the validation cohort. **m, n** Bar charts of the specificity (**m**) and accuracy (**n**) of PCaseek-D, tPSA, %fPSA, and PSAD on the validation cohort. **o** The ROC curves and corresponding AUC values of PCaseek-D, tPSA, and %fPSA for patients with gray zone PSA. **p** The ROC curve and corresponding AUC value of PCaseek-G for the classification of low GS and high GS cases in the validation cohort. For **c**, **d**, **f**‒**h**, n.s. nonsignificant, *P < 0.05, **P < 0.01, ***P < 0.001, and ****P < 0.0001 by two-sided Wilcoxon tests.

Similarly, we constructed a deep learning model named PCaseek-G based on HGS-specific pDMRs to distinguish clinically significant PCa (HGS) from indolent or insignificant PCa (LGS) before a biopsy (Supplementary Fig. S7a, b). Notably, PCaseek-G achieved AUCs of 0.99 and 0.94 to differentiate HGS patients from LGS patients on the training and validation cohorts, respectively (Fig. 1p; Supplementary Fig. S7c). Notably, the specificity and accuracy of PCaseek-G still reached 86% and 74% on the independent validation dataset (Supplementary Fig. S7d).

To avoid unnecessary biopsy and overdiagnosis, we constructed two noninvasive tests, PCaseek-D and PCaseek-G, to diagnose and grade PCa patients using urinary DNA WGBS data before a biopsy. In order to enrich tumor-related signals, we integrated DNA sequence and methylation information of the selected DMRs at a read level, and trained two deep learning models which showed effective classification outcomes. In the previously studies, the urine-based DNA methylation assays for the detection of prostate cancer yielded a suboptimal specificity^14,15^, while our model PCaseek-D exhibits an excellent diagnostic specificity especially with the low PSA levels (2‒10 ng/mL) that can be easily overlooked. Moreover, the urine DNA always needs to be extracted from digital rectal exam or first morning void. However in our study, the urinary DNA was extracted from randomly voided midstream urine, which can be obtained more easily and reliably. Certainly, there remains a risk of false-negative results of PCaseek-D/PCaseek-G leading to delayed treatment for certain cancer patients, and the combination of them with other diagnostic approaches such as multiparametric magnetic resonance imaging and/or PSMA PET-CT needs further investigation.

### Supplementary information

Supplementary information

Table S2

Table S1

Table S3

## Data Availability

The urinary DNA and tissue sequencing data in this study are deposited in the Genome Sequence Archive (GSA) for human under the accession number HRA005905 and PRJCA001124 at https://ngdc.cncb.ac.cn/gsa/.

## References

[1] Sung, H. et al. *CA Cancer J. Clin.* **71**, 209–249 (2021). 33538338 10.3322/caac.21660

[2] Dvoracek, J. *Cas. Lek. Cesk.* **137**, 515–521 (1998). 9787503

[3] Sandhu, S. et al. *Lancet* **398**, 1075–1090 (2021). 34370973 10.1016/S0140-6736(21)00950-8

[4] Sandblom, G. et al. *Cancer* **112**, 813–819 (2008). 18098207 10.1002/cncr.23235

[5] Moses, K. A. et al. *J. Natl. Compr. Canc. Netw.* **21**, 236–246 (2023). 10.6004/jnccn.2023.0014 36898362

[6] Ge, G. et al. *Clin. Chem.* **66**, 188–198 (2020). 31811000 10.1373/clinchem.2019.309633

[7] Oshi, M. et al. *Cancers* **13**, 2652 (2021). 34071230 10.3390/cancers13112652 PMC8199052

[8] Xu, Z. et al. *Eur. Urol.* **77**, 288–290 (2020). 31744643 10.1016/j.eururo.2019.11.006

[9] Chan, K. C. et al. *Proc. Natl. Acad. Sci. USA* **110**, 18761–18768 (2013).

[10] Li, W. et al. *Nucleic Acids Res.* **46**, e89 (2018). 10.1093/nar/gky423 PMC6125664 29897492

[11] Li, J. et al. *Brief. Bioinform.* **22**, bbab250 (2021).

[12] Li, J. et al. *Nature* **580**, 93–99 (2020). 32238934 10.1038/s41586-020-2135-x

[13] Ruopp, M. D. et al. *Biom. J.* **50**, 419–430 (2008). 18435502 10.1002/bimj.200710415 PMC2515362

[14] Brikun, I. et al. *Clin. Epigenetics* **10**, 91 (2018). 29988684 10.1186/s13148-018-0524-x PMC6029393

[15] Brikun, I. et al. *Exp. Hematol. Oncol.* **8**, 13 (2019). 31297302 10.1186/s40164-019-0137-x PMC6598372

