# Supplementary material for: PCaseek: ultraspecific urinary tumor DNA detection using deep learning for prostate cancer diagnosis and Gleason grading
Source: Cell Discov. 2024 Sep 3;10:90. doi: 10.1038/s41421-024-00710-y (PMC11369186; doi:10.1038/s41421-024-00710-y)
Supplement: Supplementary file 1 — Supplementary information [file 41421_2024_710_MOESM1_ESM.pdf]

Supplementary Information for

PCaseek: Ultraspecific Urinary Tumor DNA Detection Using Deep Learning for Prostate Cancer Diagnosis and Gleason Grading

Gaojie Li[1,2,3†], Ye Wang[4†], Ying Wang[1,2,3†], Baojun Wang[4†], Yuan Liang[1,2], Ping Wang[1,2,3], Yudan He[1,2,3], Xiaoshan Hu[4], Guojun Liu[4], Zhentao Lei[5], Bao Zhang[5], Yue Shi[1,2*], Xu Gao[6*], Xu Zhang[4*], Weimin Ci[1,2,3*]

† These authors contributed equally to this work.

* Correspondence:

Weimin Ci

ciwm@big.ac.cn

Xu Zhang

xzhang@foxmail.com

Xu Gao

gaoxu.changhai@foxmail.com

Yue Shi

shiyue@big.ac.cn

This file includes:

Supplementary Fig.S1 to S7

Supplementary Methods

Supplementary Figures

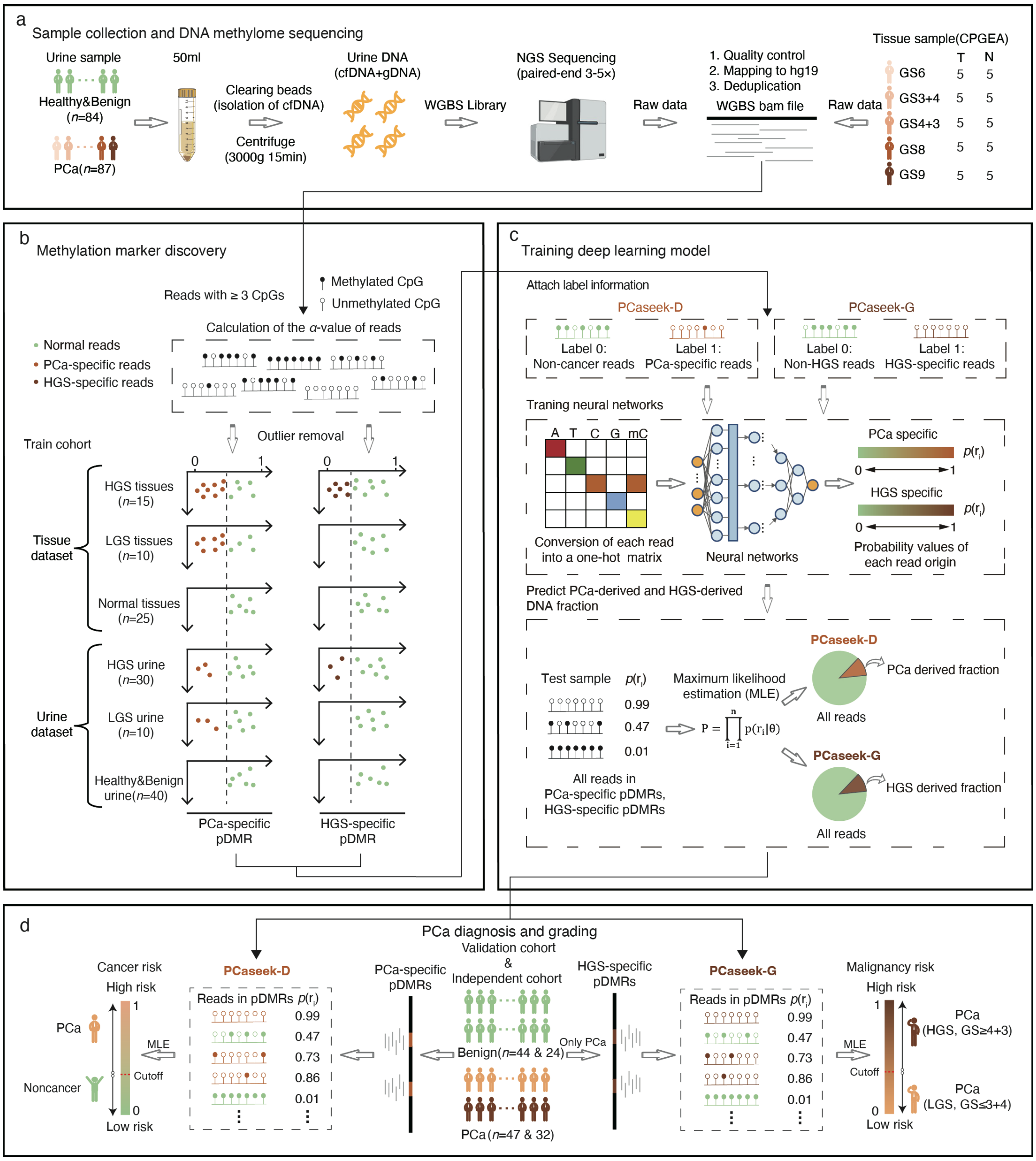

Supplementary Fig. S1. Workflow chart of data generation and PCaseek classifier development for the noninvasive detection and Gleason grading of prostate cancer. **a** Sample collection and DNA methylome sequencing. DNA methylomes by shallow whole-genome bisulfite sequencing (sWGBS) of whole urine in prostate cancer patients and healthy and benign individuals. DNA methylomes from prostate tumor tissues with various Gleason scores and matched adjacent normal tissues were obtained from the Chinese Prostate Cancer Genome and Epigenome Atlas (CPGEA) cohort. **b** Methylation marker discovery**.** Identifying PCa-specific pDMRs and HGS-specific pDMRs across the whole genome. Extraction reads from the selected pDMRs for further analysis. **c** Training deep learning model**.** Training the deep learning model with reads from pDMRs, and then employing maximum likelihood estimation to estimate the DNA fractions derived from tumor tissues and HGS tissues. **d** Diagnosis and grading of prostate cancer. For new samples, the trained neural network is used to

predict the origin of reads in pDMRs, and then to calculate the cancer risk and malignancy risk values for classification.

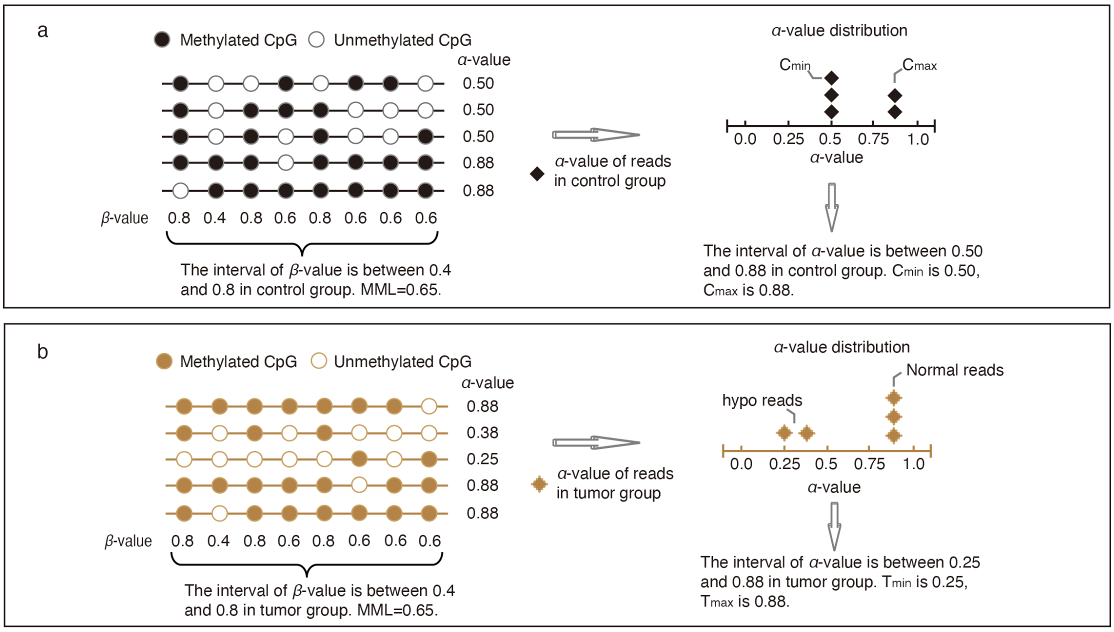

Supplementary Fig. S2. The calculation methods of the *α*-value and *β*-value within a genomic region. a For the control group, the *α*-values ranged between 0.5 and 0.88, and the *β*-values ranged between 0.4 and 0.8 with a mean methylation level (MML) of 0.65. **b** For the tumor group, the *α*-values ranged between 0.25 and 0.88, and the *β*-values ranged between 0.4 and 0.8 with a mean methylation level (MML) of 0.65. Reads in the tumor group with *α*-values between 0.5 and 0.88 were defined as normal reads, reads below 0.5 were defined as hypomethylated reads, and reads above 0.88 were defined as hypermethylated reads.

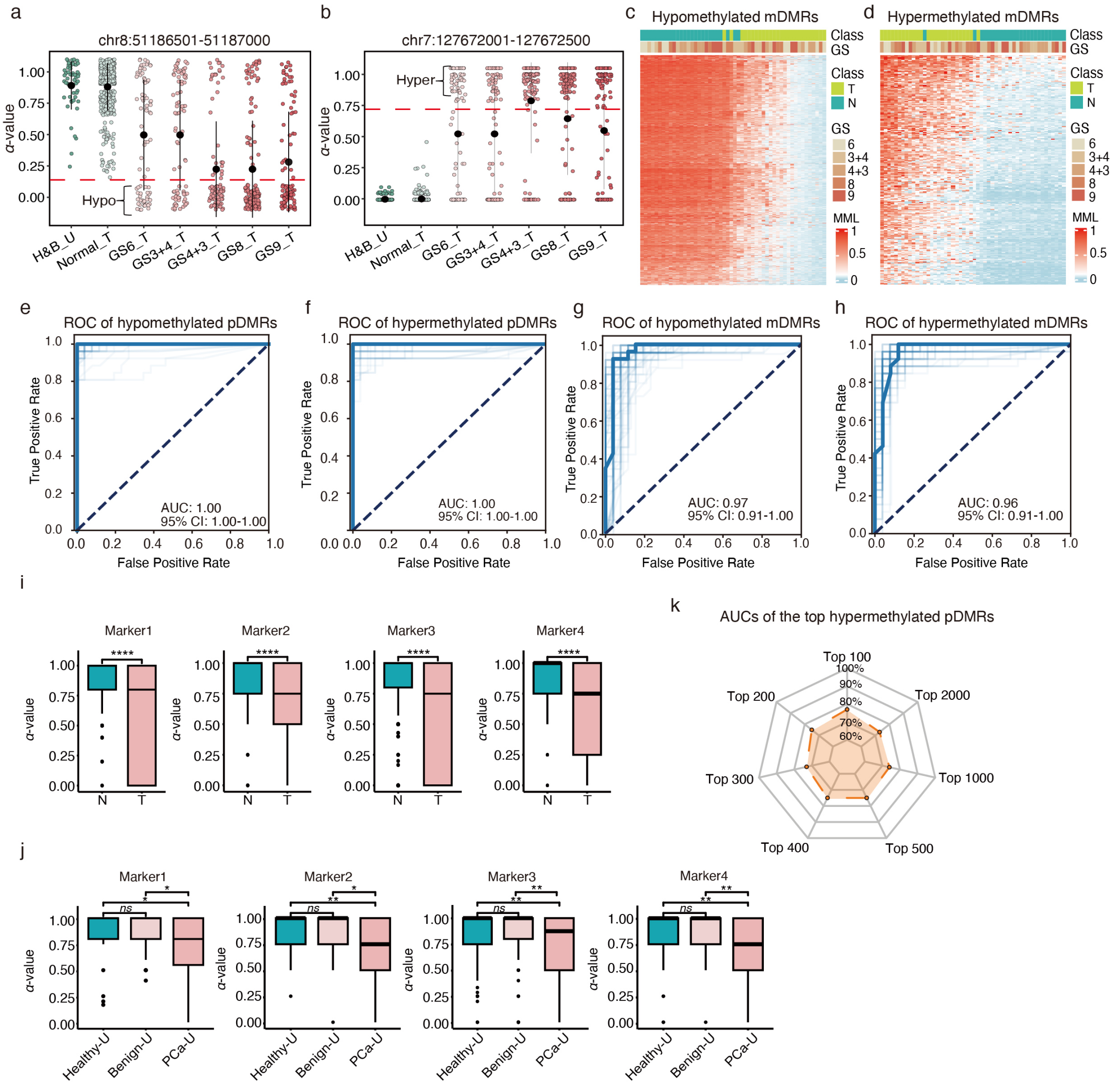

Supplementary Fig. S3. The classification performance of PCa-specific pDMRs is superior to that of PCa-specific mDMRs. a, b Scatterplots of the α-value distribution of PCa-specific hypo (chr8:51186501-51187000) (**a**) and hyper (chr7:127672001-127672500) (**b**) pDMR in healthy (n = 9, H) and benign (n = 31, B) urine samples and in normal and tumor tissues with different Gleason scores (GSs) from the CPGEA cohort. The red dashed lines represent C_{min} and C_{max}. **c, d** Heatmaps and hierarchical clustering showing the mean methylation level of the top 10% of PCa-specific hypo- (**c**) /hyper- (**d**) mDMRs between prostate cancer tissues (n = 25) and adjacent normal tissues (n = 25) in the training cohort. **e-h** ROC curves and the corresponding AUC values, reflecting the classification performance for the top 100 PCa-specific hypo- (**e**) /hyper- (**f**) pDMRs and PCa-specific hypo- (**g**) /hyper- (**h**) mDMRs. The thin curves represent the classification performance for the methylation level of the individual genomic region, and the thick curves represent the median classification performance of the methylation levels of the 100 regions. **i** Boxplots displaying the distribution of α-values of reads in four representative PCa-specific pDMRs between prostate cancer tissues (n = 25) and adjacent normal tissues (n = 25). **j** Boxplots displaying the distribution of α-values of reads in four representative PCa-specific pDMRs among

urine samples from healthy individuals (n = 9), benign prostatic disease patients (n = 31), and prostate cancer patients (n = 40). **k** Radar chart of the model performance with varying numbers of the top PCa-specific hyper pDMRs in the urine samples from the validation cohort. *n.s.* nonsignificant, *$P < 0.05$, **$P < 0.01$, ***$P < 0.001$, and ****$P < 0.0001$ by two-sided Wilcoxon tests.

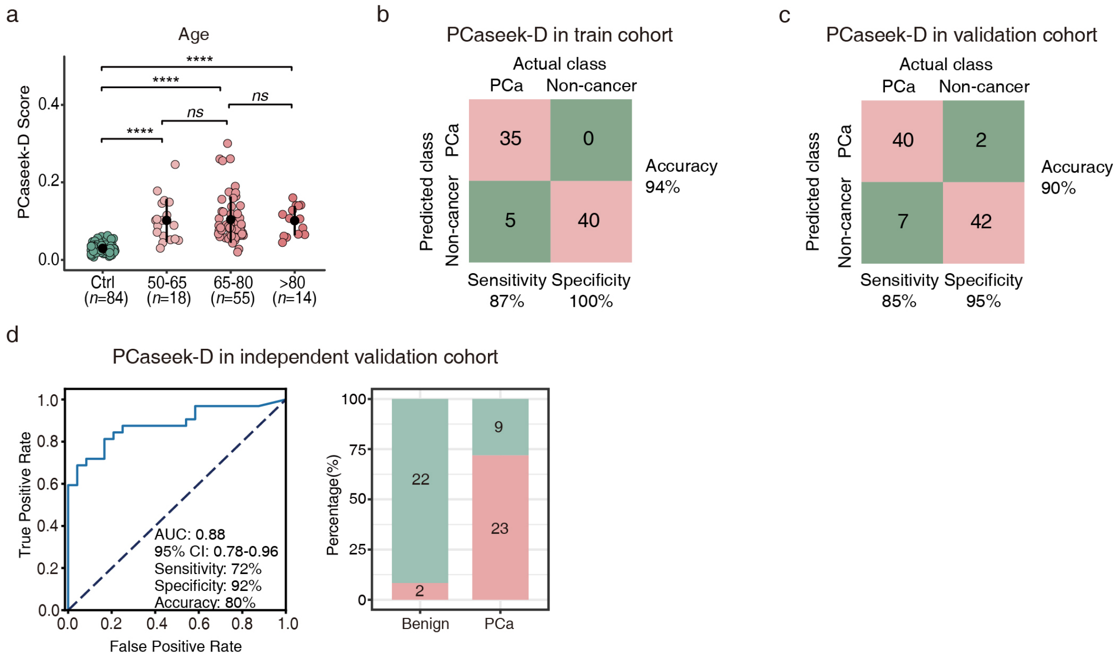

Supplementary Fig. S4. Construction and validation of PCaseek-D to detect PCa. **a** Scatterplots displaying the distribution of PCaseek-D scores across different age groups. **b, c** Confusion matrix of the PCaseek-D model on the training cohort (**b**) and the validation cohort (**c**). **d** ROC curves of the PCaseek-D model on the independent validation cohort (left). Bar chart of the specificity and sensitivity of PCaseek-D on the independent validation cohort (right). *n.s.* nonsignificant, **P* < 0.05, ***P* < 0.01, ****P* < 0.001, and *****P* < 0.0001 by two-sided Wilcoxon tests.

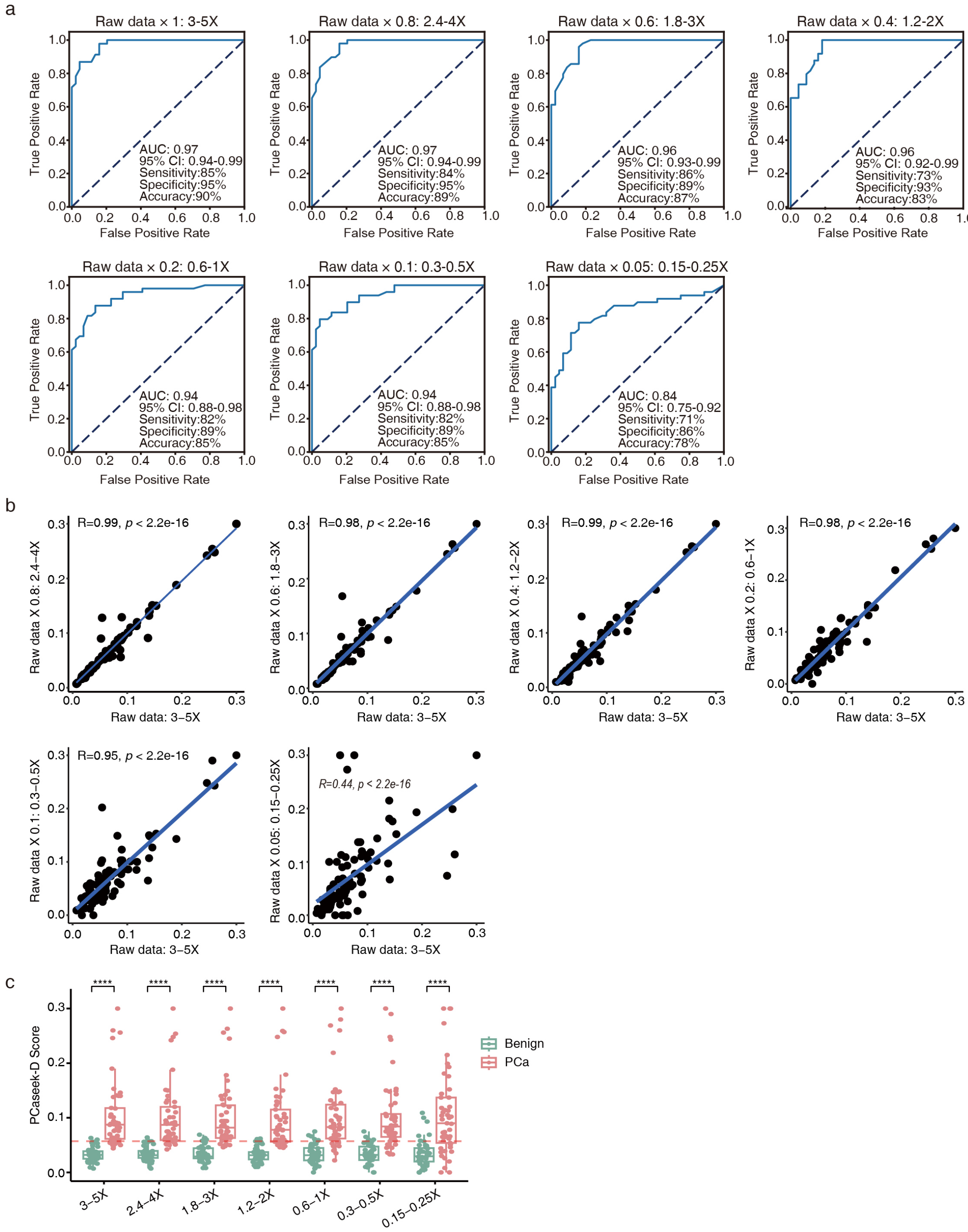

Supplementary Fig. S5. The PCaseek-D model maintains good classification performance with low-depth sequencing data. **a** ROC curves of the PCaseek-D model at different sequencing depths on the validation cohort. **b** Scatter plots showing the Pearson correlation coefficients of the PCaseek-D scores between the low sequencing depth and the original sequencing depth. **c** Boxplots of PCaseek-D scores at different sequencing depths, with the red dashed line indicating the cutoff of PCaseek-D.

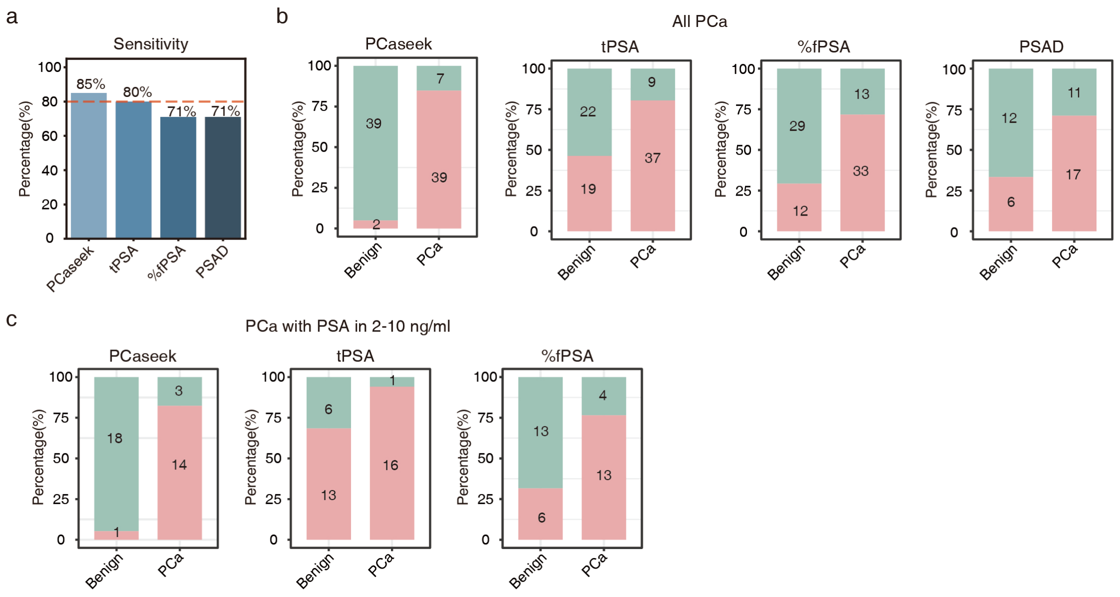

Supplementary Fig. S6. The classification performance of the PCaseek-D model is superior to the clinical indicators tPSA, %fPSA, and PSAD. a Bar chart of the sensitivity of PCaseek-D, tPSA, %fPSA, and PSAD on the validation cohort. **b** Stacked bar charts comparing the classification performance of the PCaseek-D model with the clinical indicators, including tPSA, %fPSA, and PSAD. **c** Stacked bar charts depicting the classification performance of the PCaseek-D model, tPSA, and %fPSA in samples within the PSA gray zone (2–10 ng/mL).

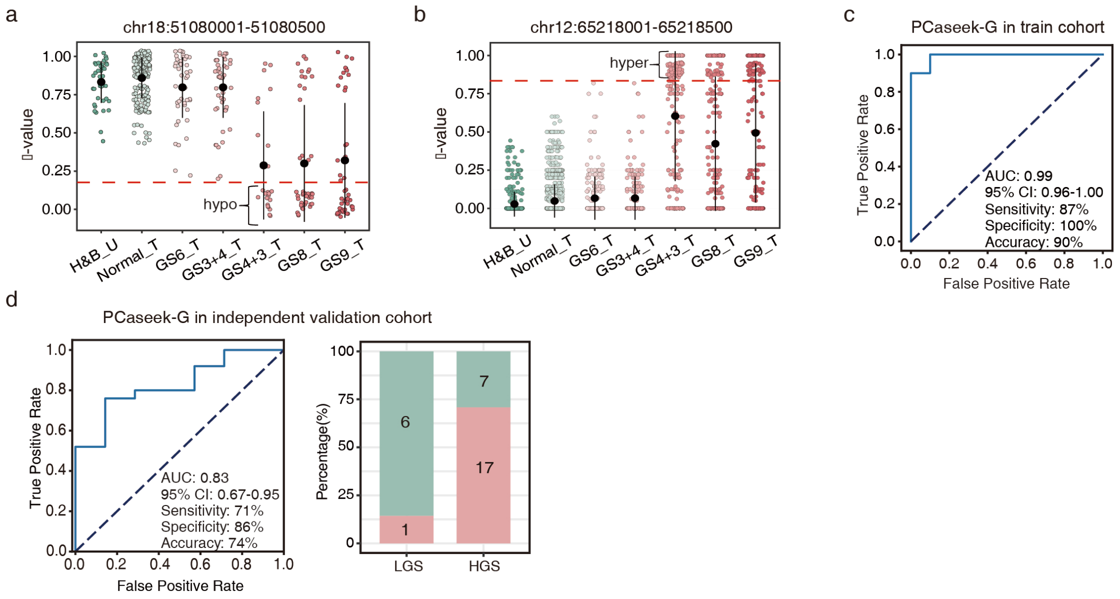

Supplementary Fig. S7. Construction and validation of PCaseek-G. a, b Scatterplots of the *α*-value distribution of HGS-specific hypo (chr18:51080001-51080500) (**a**) and hyper (chr12:65218001-65218500) (**b**) pDMRs in healthy ($n = 9$, H) and benign ($n = 31$, B) urine samples and in normal and tumor tissues with different Gleason scores from the CPGEA cohort. The red dashed lines represent C_{min} and C_{max}. **c** The ROC curve and corresponding AUC value of PCaseek-G for the classification of low GS and high GS cases in the training cohort. **d** ROC curves of the PCaseek-G model on the independent validation cohort (left). Bar chart of the specificity and sensitivity of PCaseek-G on the independent validation cohort (right).

Supplementary Methods

Overview

The ultimate goal of PCaseek is to diagnose and grade prostate cancer by integrating DNA sequence and methylation information in urinary cell-free and genomic DNA methylation sequencing data by inferring the sequencing read origin and enhancing the cancer-related signal at the read-resolution. In this study, the overall process can be divided into 4 steps: **(1) Sample collection and data generation.** WGBS data were obtained from urine samples, including prostate cancer patients, benign prostatic disease patients (benign prostatic hyperplasia (BPH), prostatitis), and healthy individuals, and from tissue samples, including Gleason score (GS) 6 to GS9 tumor tissues and adjacent normal tissues, from the CPEGA cohort in our previous research (Supplementary Fig. S1a)[1]. **(2) Identification of differentially methylated regions (DMRs) specific to prostate cancer and risk stratification.** PCa and high Gleason score (Gleason score ≥ 4+3, HGS)-specific pDMRs refer to regions where there is a significant difference in the α-value in both tissue and urine samples from tumor patients and noncancer individuals. These identified pDMRs are considered candidate biomarkers for model construction (Supplementary Fig. S1b). **(3) Extract reads from the selected pDMRs to train the deep learning model.** Train a deep learning model to integrate DNA sequence and methylation information. The output of the models PCaseek-D and PCaseek-G is a probability value indicating the likelihood that a read originates from tumor tissues and HGS tissues, respectively (Supplementary Fig. S1c). **(4) Diagnosis and grading of prostate cancer.** For a new sample, PCaseek-D can classify each read into either the tumor-derived DNA class or the nontumor-derived DNA class and then estimate the proportion of tumor-derived reads in the whole urinary DNA. PCaseek-G estimates the fraction of reads derived from HGS tissues to infer whether the PCa patient is suffering from aggressive prostate cancer (Supplementary Fig. S1d). The more reads enriched, the higher the risk of the sample. The detailed individual steps are in the sections below.

Ethics approval

The study was approved by the Ethics Committee of the Chinese PLA General Hospital (No. 2023(020)), Changhai Hospital (CHEC2019-012), and Aerospace Center Hospital (No. 2023(121)). Additionally, this study was registered on ChiCTR.org (ChiCTR2200059901). The procedures used in this study adhered to the tenets of the Declaration of Helsinki, and all patients provided written informed consent prior to enrollment.

Sample collection and data generation

Urine samples were collected from PCa patients and benign prostatic disease patients at Chinese PLA General Hospital (CPLAGH, $n_{tumor} = 92$, $n_{benign} = 61$) and Aerospace Center Hospital (ASCH, $n_{tumor} = 27$, $n_{benign} = 38$) before operations, as well as from 9 healthy volunteers without any diseases, with participant ages ranging from 18 to 95 years old. In this study cohort, PCa patients and benign prostatic disease patients were needed to have tissue samples clearly obtained through biopsy for pathological

assessment before being included in the study. The healthy volunteers successfully passed a physical examination indicating the absence of prostate disease. All surgically resected prostate samples were evaluated by two pathologists, and the results were obtained through pathological consultation. All PCa patients, ranging from stages GS6 to GS10 according to the Gleason score of their pathological tissues. All participants were properly informed about the study and signed the informed consent. In the experiment, all samples were deidentified and reencoded for patient privacy protection.

The CPGEA data were downloaded from the Genome Sequence Archive for Human (http://bigd.big.ac.cn/gsa-human/) at the BIG Data Center, Beijing Institute of Genomics, Chinese Academy of Sciences, under accession number PRJCA001124[1]. Detailed information can be found in Supplementary Table S1.

We estimated the minimum sample sizes based on the preset sensitivity and specificity. The calculations are as follows.

$$N_{Tumor} = \frac{(Z_{1-\frac{\alpha}{2}} * \sqrt{p_{se} * \ 1 - p_{se}}\ ^{2}}{\delta_{se}^{2}}$$

$$N_{Benign} = \frac{(Z_{1-\frac{\alpha}{2}} * \sqrt{p_{sp} * \ 1 - p_{sp}}\ ^{2}}{\delta_{sp}^{2}}$$

In the above formula, $\alpha = 0.05$, δ_{se} and δ_{sp} are both 0.1, $p_{se} = 0.85$, and $p_{sp} = 0.90$. After calculation, a minimum of 49 urine samples from PCa patients and 35 urine samples from benign prostatic disease patients should be collected. In this study, we collected a total of 99 urine samples from benign prostatic disease patients, 9 from healthy individuals, and 119 from PCa patients, which meets the requirement of "minimum sample size". Detailed information can be found in the Supplementary Table S2.

DNA extraction and WGBS library construction

Before urine collection, urine conditioning buffer was added to the tube to prevent the degradation of cellular and cell-free DNA. Urine (50 mL) without the need for digital rectal examination (DRE) was collected and stored at -80 °C within 30 min. Whole DNA (cellular and cell-free DNA) from urine was isolated using the Quick-DNA Urine Kit (Zymo Research, catalog No. D3061) according to the manufacturer’s instructions. In brief, clearing beads were used to bind cell-free DNA from each urine sample, followed by centrifugation at 3000 g for 15 minutes. A total of 50–400 ng of urinary DNA per sample was used for whole-genome bisulfite sequencing (WGBS) library construction as described before[2, 3].

Sequencing and mapping

The libraries from all samples were sequenced on NovaSeq 6000 sequencers (Illumina) to generate 150 bp paired-end reads (sequencing depth 3X–5X). We utilized fastp (version 0.23.4) to trim the sequencing adapters and low-quality data from the raw paired-end sequence data and obtained clean data for subsequent analysis[4]. Next, the

clean data were aligned to the hg19 version of the human genome using BS-Seeker2 (version 2.1.8) in pair-end mode (parameters: -aligner Bowtie2 –XS 0.5,5)[5, 6]. Reads that aligned to multiple regions of the genome were discarded simultaneously, and only those reads with unique mappings were retained. SAMtools (version 1.9) and sambamba (version 1.0.0) were then employed to sort and remove PCR duplication reads[7]. Depth and coverage statistics were generated using mosdepth (version 0.3.4), and the conversion rate of the sample was evaluated using pysam (version 0.15.3)[8].

Identification of PCa-specific DMRs and HGS-specific DMRs

In previous studies, DMRs were selected by comparing the differences, such as the mean methylation level or the maximum and minimum values, in two or more sets of groups[9, 10]. However, comparing interval mean methylation levels may obscure differences between distinct cell types, making it inadequate for sensitivity. Simultaneously, outliers may significantly influence the maximum and minimum values within an interval, failing to reflect the overall methylation status accurately. To mitigate the impact of these factors and identify more stable and reliable DMRs, we proposed a method based on the **p**roportion of aberrant methylation reads to identify PCa (HGS)-specific pDMRs.

Simultaneously, to ensure the representativeness and diversity of the training cohort, we adopted a random sampling method based on Gleason score and randomly selected 25 pairs of tumor tissues and matched adjacent normal tissues from the CPEGA cohort. For urine samples, we randomly chose samples from 40 PCa patients and 31 benign prostatic disease individuals. Furthermore, to enhance the complexity of the training cohort, we intentionally included all samples from 9 healthy individuals. The remaining urine samples were used as a validation cohort.

Identifying pDMRs

Step 1: Calculation of the α-value for each genomic region. The human genome was divided into nonoverlapping regions of 500 bp, resulting in 6,191,368 regions. We calculated the α-value for reads that contained three or more CpG sites in each region (Supplementary Fig. S2). In this step, the α-value distribution was obtained for each region and used for the subsequent selection of DMRs.

Step 2: Removal of outlier α-values. In tumor tissues, in addition to tumor cells, there are also various nontumor cells, such as immune cells and vascular endothelial cells. Additionally, urinary DNA methylation may be influenced by individual differences and environmental factors. Therefore, to better represent the methylation levels of each group, we calculated the Z-score for the α-value of each read to determine outliers, and the reads with $|z| > 3$ were removed. In this step, outlier α-values were removed, and the minimum and maximum values within each genomic region were adjusted to approach the true distribution of each group.

Step 3: Identification of PCa-specific pDMRs and HGS-specific pDMRs. First, we merged the WGBS data from noncancer urine samples and adjacent normal tissues. Then, genomic regions with sequencing depths below 50X were removed from the merged urine samples to ensure sufficient read coverage. We used C_{min} and C_{max} to represent the minimum and maximum α-values, respectively, of a region in the control

group (adjacent normal tissues, benign and healthy urine samples). Therefore, all α-values for reads in normal tissues and noncancer urine samples should fall between C_{min} and C_{max}. We defined the reads with α-values > C_{max} as hypermethylated reads and reads with α-values < C_{min} as hypomethylated reads in tumor tissues. The proportion of hypo(hyper)methylated reads among all reads was represented by the term P-score. Hypo-pDMR was selected as an example, and the P-score was calculated as follows:

$$\text{P-score} = \frac{\text{count}\left(\alpha\text{-values} < C_{min}\right)}{\text{reads count}}$$

To achieve good sample complexity for the performance of the model with different Gleason scores, we chose tumor tissues with different GSs (a total of 5 groups: GS6, GS3+4, GS4+3, GS8, and GS9) and merged the data. The **s**um of P-scores for all GS groups is referred to as the SP-score. We identified genomic regions with a P-score ≥ 0.15 and an SP-score ≥ 1 as PCa-specific pDMRs. In addition, these differences were present in both urine samples from PCa patients and noncancer urine samples (Supplementary Fig. S3a, b; Fig. S3i, j and Table S3). Moreover, the identification process of HGS-specific pDMRs is similar to that of PCa-specific pDMRs (Supplementary Fig. S7a, b). The identified PCa-specific pDMRs and HGS-specific pDMRs can distinguish between tissue and urine samples from PCa patients and noncancer individuals, as well as from patients with high GS and low GS.

Identifying mDMRs

To discover the optimal methylation biomarkers for PCa, we utilized the traditional method of **m**ean methylation levels to identify DMRs, termed PCa-specific mDMRs (Supplementary Fig. S2). The objective was to compare the stability and reliability of pDMRs with mDMRs. The calculation process is as follows:

$$\text{M-score} = \text{Tumor}\left(\frac{C}{C+T}\right) - \text{Control}\left(\frac{C}{C+T}\right)$$

C represents the count of methylated CpG in one genomic region, and T represents the count of unmethylated CpG. Similar to the SP-score, the SM-score represents the sum of M-scores across all GS groups. We identified genomic regions with an M-score of ≥ 0.15 and an SM-score of ≥ 1 as PCa-specific mDMRs.

Here, we obtained two types of DMRs (pDMRs and mDMRs) on the basis of two different signatures and sorted these DMRs in descending order based on the SP-scores and SM-scores. The higher the score is, the more tumor signals from the specific genomic region are released, and the more detection is likely.

Training a deep learning model to predict the source of reads

In previous studies, a cutoff was set to select a certain number of DMRs for model training[9, 10]. However, there is significant variation between different cancers, and a cutoff set for one cancer may not be suitable for another. At the same time, each pDMR releases a certain proportion of tumor signals, and the choice of the number of differential regions directly impacts the performance of the model. Opting for too few differential regions may result in the model's inability to capture sufficient tumor signals, thereby reducing the sensitivity. Conversely, selecting too many differential regions

may introduce noise and low-quality data, affecting the specificity and overall performance of the model. Therefore, before constructing the model, we first assessed the number of pDMRs that impact the performance of our classifiers. Therefore, we adopted an approach for selecting different numbers of pDMRs based on their quantities and evaluated the classification performance on the validation cohort. The deep learning model mainly referred to the DISMIR deep learning network architecture, which primarily consists of convolutional neural networks (CNNs) and bidirectional long short-term memory (BiLSTM) layers. The workflow for constructing the deep model is as follows:

Step 1: Attach label information based on the source of the reads

For the PCaseek-D model, we extracted the PCa-specific reads (labeled "1") from tumor tissue, as well as noncancer reads (labeled "0") from normal tissues and noncancer urine samples. For the PCaseek-G model, we extracted the HGS-specific reads (labeled "1") from HGS tissues, as well as non-HGS reads (labeled "0") from LGS tissues and urine samples of non-HGS patients. We removed the first and last 5 bp from each read to eliminate the influence of sequencing adapters and base quality degradation. Finally, reads of approximately 140 base pairs in length were obtained for subsequent model construction.

Step 2: Convert each read's bases into a one-hot matrix representation. In this representation, A bases are represented as (1, 0, 0, 0, 0), T bases as (0, 1, 0, 0, 0), C bases as (0, 0, 1, 0, 0), G bases as (0, 0, 0, 1, 0), and 5mC bases as (0, 0, 1, 0, 1).

Step 3: Input the matrix with label information to the deep learning model for training. This neural network comprises one-dimensional convolutional layer, max pooling layer, dropout layer, bidirectional long short-term memory (BiLSTM) layer, and a fully connected layer. Features were extracted through a series of convolution and pooling operations, and sequential information was processed by bidirectional LSTM. Finally, the sigmoid function was used as a binary classification output layer to output a classification probability value between 0–1. The larger the value is, the more likely it is from the tumor tissue or HGS tissue.

This section primarily involves training a deep learning model named PCaseek for inferring the origin of reads, thereby achieving the enrichment of tumor (HGS)-derived reads in urine. The details of the deep learning model are illustrated in Supplementary Fig. S1c.

Calculation of the cancer risk and malignancy risk by estimating the proportion of tumor-derived and HGS-derived DNA

As illustrated in Supplementary Fig. S1d, we use PCaseek-D to predict the source of reads within the selected methylation marker regions for a new sample. Subsequently, we calculate the cancer risk that the sample is PCa using reads from selected PCa-specific pDMRs. Similarly, we determine the malignancy risk of the PCa patient being high grade cancer by using reads from selected HGS-specific pDMRs. Here, cancer risk is presented as an example. The risk values are calculated using the method of maximum likelihood estimation (MLE), and the calculation process is as follows:

$$\begin{cases} P = \sum_{i=1}^{n}\left[\theta \times p_i + (1-\theta)\times\left(1-p_i\right)\right] \\ \hat{\theta} = \arg\max_{\theta}\left(\sum_{i=1}^{n}\left[\theta \times p_i + (1-\theta)\times\left(1-p_i\right)\right]\right) \end{cases}$$

Here, n represents the number of reads from selected methylation PCa-specific pDMRs; p represents the probability value generated by the above trained deep learning model, indicating the likelihood that reads originate from tumor tissue; θ represents the proportion of tumor reads in methylation marker regions, and our goal is to find a parameter θ to maximize the value of P. Since the θ value is between 0 and 1 and the parameter space is relatively small, we employ an exhaustive search method with a step size of 0.001 to estimate θ, aiming to identify the parameter values that maximize the posterior probability. A larger θ value indicates a higher proportion of tumor-derived reads, making the new sample more likely to be from a cancer patient. The calculation methods for malignancy risk are similar to those for cancer risk.

Youden index and optimal cutoff estimated

After model construction, the Youden index was used to select the optimal cutoff for improving the sensitivity and specificity of tumor detection. Youden index (J) can be formally defined as follows:

$$J = \max_{C}\left\{Se\left(c\right) + Sp\left(c\right) - 1\right\}$$

The cutoff that achieves this maximum is called the optimal cutoff (c*) because it optimizes the biomarker's differentiating ability when sensitivity and specificity are weighted equally[11, 12].

Model evaluation

The final models PCaseek-D and PCaseek-G were obtained through training and validation cohorts. To further evaluate the performance of the models, we subsequently collected urine samples from PCa patients and benign prostatic disease patients at CPLAGH as an external independent validation set to further assess the classification performance of the models. Evaluation metrics for the models include accuracy, sensitivity, specificity, true positives (TP), true negatives (TN), false positives (FP), false negatives (FN), receiver operating characteristic (ROC) curve, and the area under the ROC curve (AUC) values were calculated[13].

Statistical analysis

The statistical analysis and data visualization used in this study were performed by using R version 4.3.0 and Python version 3.10.5. The R packages used include, but are not limited to, “devtools”, “dplyr”, “ggplot”, “ggpubr”, and “ComplexHeatmap”. The Python packages used include, but are not limited to, “numpy”, “pandas”, “matplotlib”, “scipy”, “pysam”, “sklearn”, “keras”, and “TensorFlow”. The optimal threshold was evaluated by the ROC curve. The Youden index was used to select the optimal threshold for improving the sensitivity and specificity. All hypothesis tests were two-sided, and

$P < 0.05$ was considered statistically significant.

References:

1. Li, J. *et al. Nature* **580**, 93-99 (2020).
2. Wang, P. *et al. EBioMedicine* **89**, 104437 (2023).
3. Xu, Z. *et al. Eur Urol* **77**, 288-290 (2020).
4. Chen, S. *et al. Bioinformatics* **34**, 884-890 (2018).
5. Guo, W. *et al. BMC Genomics* **14**, 774 (2013).
6. Langmead, B. *et al. Genome Biol* **10**, R25 (2009).
7. Li, H. *et al. Bioinformatics* **25**, 2078-2079 (2009).
8. Pedersen, B.S. & Quinlan, A.R. *Bioinformatics* **34**, 867-868 (2018).
9. Li, W. *et al. Nucleic Acids Res* **46**, e89 (2018).
10. Li, J. *et al. Brief Bioinform* **22**, bbab250 (2021).
11. Fluss, R. *et al. Biom J* **47**, 458-472 (2005).
12. Ruopp, M.D. *et al. Biom J* **50**, 419-430 (2008).
13. Zweig, M.H. & Campbell, G. *Clin Chem* **39**, 561-577 (1993).
